# Lower-dose psycholytic therapy – A neglected approach

**DOI:** 10.3389/fpsyt.2022.1020505

**Published:** 2022-12-02

**Authors:** Torsten Passie, Jeffrey Guss, Rainer Krähenmann

**Affiliations:** ^1^Hannover Medical School, Hannover, Germany; ^2^Dr. Senckenberg Institute for History and Ethics of Medicine, Goethe University Frankfurt, Frankfurt, Germany; ^3^Grossman School of Medicine, New York University, New York, NY, United States; ^4^Fluence International, Inc., Woodstock, NY, United States; ^5^Psychiatric Services Thurgau, Münsterlingen, Switzerland; ^6^University Hospital for Psychiatry and Psychotherapy, Paracelsus Medical University, Salzburg, Austria; ^7^Department of Psychiatry, Psychotherapy and Psychosomatics, University of Zurich, Zürich, Switzerland

**Keywords:** psycholytic therapy, psychoanalysis, hallucinogens, mystical experience, LSD, psilocybin, MDMA, lower-dose LSD therapy

## Abstract

Lysergic acid diethylamide (LSD) and similar psychoactive drugs have been used in psychotherapy since 1949, when the first clinical study with lower-dose LSD showed therapeutically relevant effects. This caused an intense interest among psychotherapists and researchers, alike, on an international scale. In 1960, the use of serial lower-dose LSD/psilocybin sessions in a psychoanalytical framework, which was dominant at the time, was named *“psycholytic therapy”*. Psycholytic therapy was usually conducted in clinical environments, on both an inpatient and outpatient basis. Psycholytic therapy was developed and established over a 15-year period on the European continent, where it was used at 30 clinical treatment centers and by more than 100 outpatient psychotherapists. Psycholytic approaches were employed minimally in North America, where the *psychedelic approach* (use of one or two high-dose sessions for “personality-transforming mystical experiences”) became the dominant method in use. The leading figure in psycholytic therapy was Professor Hanscarl Leuner in Germany, who laid the ground with his uniquely fine grained analysis of the LSD reaction in a 1962 monograph. He was central in establishing and distributing psycholytic therapy in Europe and abroad. The article provides comprehensive background information and outlines the essential features of psycholytic therapy. Evidence for the efficacy of psycholytic therapy is reviewed and a case for the inclusion of the psycholytic approach in the field of substance-assisted psychotherapy is made.

## Introduction

Recently, after an interruption in research of more than 30 years, a “psychedelic renaissance” (1) has emerged, that is a revival of research and therapeutic studies with psychedelic drugs.

During the late 1950s, Abram Hoffer and Humphrey Osmond, two Canadian psychiatrists, developed the psychedelic treatment technique (2). This procedure made induction of mystic-religious experiences the basis of its therapeutic action. It uses a specific preparation of the patient, high to very high dosages, specific surroundings, and music to favor evocation of transformational insights and mystical experiences. Psychedelic treatment was developed and used during the 1960’s in a few clinics in North America. Notably, none of the controlled efficacy studies showed significant results in favor of psychedelic peak therapy for alcoholism (3–7), or chronic neuroses (8). More recent studies, however, suggest that better results can be reached (9, 10), especially with patients treated for their anxiety and depression induced by a life-threatening somatic disease (11–15). However, most of these patients had a healthier personality make-up at baseline, a better base to activate salutogenetic forces by psychedelic peak therapy.

From an overview of the last ten years, we observe that recent RCT studies are solely focused on the psychedelic approach. The appeal of this seemingly rapid, novel and impressive treatment and the much greater time required for psycholytic therapy may help explain why psycholytic therapy is underutilized and undertheorized in the contemporary psychedelic research scene (16). With this as background, we offer education about *psycholytic therapy*. We invite consideration of the vital and extensive history of psycholytic work, in particular the work done by its most central figure, Professor Hanscarl Leuner (1919-1996). Leuner worked at the University of Göttingen in Germany, on the foundations of psycholytic therapy and headed the *European Medical Society for Psycholytic Therapy (EPT)* as its president. We include basic research, methods, findings and concepts developed by researchers and analytically oriented clinicians using psychedelics to facilitate psychotherapy.

Relevant disclosures: the first author (TP) worked clinically with Hanscarl Leuner for three years. The last author (RK) is Medical Director of a 280 bed psychiatric hospital in Switzerland, the Münsterlingen Psychiatric Hospital, where he has supervised the use of lower-dose psycholytic therapy for treatment-resistant patients with anxiety and depression syndromes. A modified model of psycholytic therapy is currently an approved treatment approach in Switzerland in a compassionate use program (17).

### The history of psycholytic therapy

In the 1920s, researchers provided extensive clinical description of the mescaline intoxication (18), but most researchers at the time did not find that the experiences reflected the psychodynamics of their experimental participants [cf. (19)]. In contrast, the first systematic clinical experiments utilizing lower doses of LSD (10-50 mcg) in “psychologically-minded” participants revealed the usefulness of LSD in evoking psychodynamic material (20). Many psychoanalysts followed these observations and began to utilize LSD assisted psychoanalytic sessions in order to evoke psychodynamic processes, e.g., softening of defenses, regression, deepening of transference, spontaneous childhood memories (21–26). Observations of two authors were key for the development of the psycholytic approach. In 1953, the British psychiatrist Ronald A. Sandison worked with LSD in non-therapeutic experiments. He described intensification of affect and abreactive memory actualizations which led to a marked improvement in symptoms for some experimental subjects (22). In 1953, the German psychiatrist Hancarl Leuner developed a day-dream technique for use in psychotherapy, which he termed “Guided Affective Imagery” (27). He found that the use of small doses of LSD lead to enhanced generation of images and emotions, and that the therapeutic process was deepened, including experiences of regression and catharsis (24). In his extensive basic research on the LSD reaction, overseeing and carefully evaluating more than 1,000 experimental LSD sessions, he found proof of psychodynamically relevant experiences and their basis in biographical history of the patient (25). He also was able to outline the principal mechanisms for LSD’s therapeutic action in the framework of psycholytic therapy (25). His fine-grained analysis of the LSD reaction in his 300-page monograph is one of the most comprehensive psychological analysis of the LSD reaction up to today. Unfortunately, Leuner’s monograph as well as all his later research until the 1990s has been published just in German. This contributed to the neglect of psycholytic therapy on an international scale. Fortunately, a comprehensive English monograph on the work of Leuner will be issued in 2023 (28).

The psycholytic method was developed in the conventional context of the psychoanalytic therapies which supported the activation of memories, emotional states, and focused on the psychological conflict which was usually centered in the drive/defense/conflict model of treatment that was prevalent at the time. The use of repeated, small LSD sessions in the context of psychodynamic therapy was termed *psycholytic therapy* (*psycholysis* in short) by Ronald Sandison at the *First European Symposium for Psychotherapy under LSD-25* in 1960 (29). It should be mentioned here that in psycholytic therapy, as with all psychotherapeutic work, it is not assumed that the memories of the patients represent historical truth, but they contain emotionally meaningful content. Memories recovered during altered states of consciousness are often distorted and bizarre (30) and must not be taken as historical anamnesis (31).

The best known writers and practitioners of psycholytic therapy were: Ronald Sandison (UK), Hanscarl Leuner (Germany), Walter Frederking (Germany), Einar Geert Jörgensen (Netherlands), Stanislav Grof (Czechoslovakia, USA), Milan Hausner (Czechoslovakia), John Buckman (UK, USA), Joyce Martin (UK), Cornelius van Rhijn (Netherlands), Harold Abramson (USA), Charles C. Dahlberg (USA), and Claudio Naranjo (Chile/USA) [cf. (32)].

A review of the academic literature on psycholytic and psychedelic therapy reveals that *psycholytic* therapy was applied almost exclusively in Europe, whereas *psychedelic* therapy was more prominent in North America (Canada and USA) [cf. (33)]. Reasons for this are likely to lie in historical and cultural factors, which are outside the scope of this paper. Table 1 provides an overview of essential characteristics of the two methods.

**TABLE 1:** Comparison of the two main methods of using hallucinogens in psychotherapy [cf. (4, 39)].

	Psycholytic therapy	Psychedelic therapy
Dosage	Lower doses [e.g., LSD 30-150 mcg, psilocybin 3-15 mg]	High doses (LSD 250-800 mcg, psilocybin 25-40 mg)
Intended effects	Activation of the primary process Dream-like symbolic imaginings, regressions, transference phenomena Spontaneous insights Affective arousal and vividness of conscious experience	Cosmic-mystical “peak experience” Ecstatic experience Self-transcendence Awe Connection to others and to self
Theoretical foundation	Psychoanalytic theory Psychodynamic theories	Foundations in religions and philosophical concepts Transpersonal psychology
Number of sessions	Numerous sessions required (5-25)	1-2 session(s)
Therapeutic procedure	Psychodynamic preparation Analytical discussion of experiential material in individual and group sessions	Strongly suggestive preparation Use of specific environmental conditions and structuring music Reduction in focus on language, narrative creation and meaning making
Ego-functions	Ego functions softened Some ego functions altered while others left intact Ego boundaries softened, permeable but essentially retained Capacity for self-reflection (“reflecting ego remnant”) is maintained	Narrative identity is removed from awareness Ego-dissolution (loss of defenses) leads to experience of mystical experience, flow, cosmic consciousness, etc. Self-reflective ego is dissolved, though memory maintained
Therapeutic processing	Facilitation of familiar psychotherapeutic processes: deeper emotional truth, greater self honesty and acceptance Reality adjustment Stimulation to integrate experiences into everyday life	Narration and affirmation of peak experience Reality adjustments will follow naturally from peak experience Psychotherapeutic processing not relevant to experience
Therapeutic goal	Healing through restructuring of the personality in a maturation process Release of infantile parental bonds Change of attachment style	Direct experience of awe, sacredness, the numinous, etc. Self-transformation through change in self-experience and perspective “Spiritual awakening”
Indications	Historical: Classical indications of psychotherapy: neuroses, psychosomatic cases, personality disorders, sexual deviations Contemporary: Same as above	Historical: Alcoholism, terminal patients, drug addiction Contemporary: Cancer related Existential Distress and Demoralization Syndrome and End of life care for individuals in palliative treatments, depression, alcoholism, cigarette addiction, obsessive-compulsive disorder, posttraumatic stress disorder

From 1960 to 1975, numerous congresses and conferences were held at which psycholytic therapists presented their procedures, cases and results [cf. (32–34)]. By the mid-1960s, psycholytic therapy was a promising and widely applied approach, being conducted at more than 30 European clinics and by more than 100 outpatient therapists (32, 35). Out of the need for community as well as codifying and teaching psycholytic therapy, the *European Medical Society for Psycholytic Therapy (EPT)* was founded in 1965. The EPT conducted 5 symposia until its dissolution in 1974 (32). During these years, psycholytic therapy was refined and established as a respected clinical method [cf. (34)].

Psycholytic therapy did not come to an end due to dangers or lack of efficacy. The end of the 1960s saw increasing prohibition of psychedelic research, especially in the USA, and the subsequent laws enacted by the US Drug Enforcement Administration and in the sequel by the United Nations led to the cessation of research with LSD and similar psychoactives. Therefore, the expansion of psycholytic therapy was no longer possible. In spite of these realities, two professors central to the psycholytic therapy (Hanscarl Leuner in Germany and Jan Bastiaans in Netherlands) retained their licenses until their retirement in the mid-1980s, and continued their work. And between 1988 and 1993, five Swiss psychiatrists were granted exemption licenses for the use of 3,4-Methylenedioxymethamphetamine (MDMA) and LSD in psycholytic therapies [cf. (36)].

## Substances used with psycholytic therapy

**FIGURE 1:**
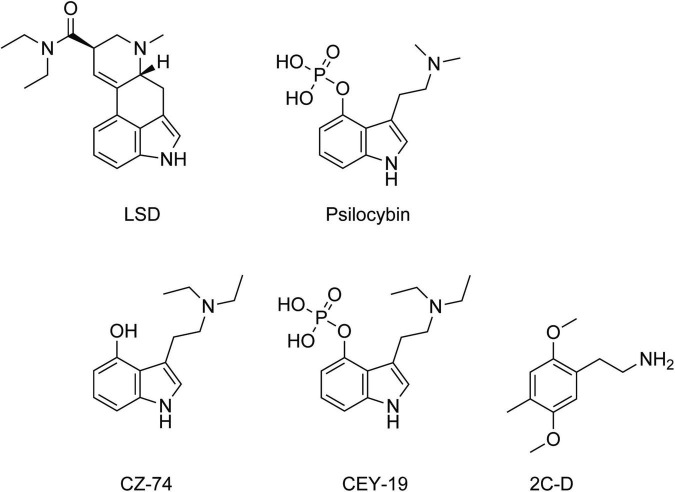
Chemical structures of the main psycholytic substances lysergic acid diethylamide (LSD), psilocybin, the psilocin derivatives CEY-19, CZ-74, and the phenethylamine 2C-D.

The primary substances used in psycholytic therapy in the 1960s were LSD and psilocybin and their congeners. The general term for these substances was “psycholytic drugs” or “psycholytics.” Around 1960, Sandoz developed two short-acting derivatives of psilocybin, codenamed CZ-74 (4-HO-DET, 4-hydroxy-N,N-diethyltryptamine) and CEY-19 (ethocybin, 4-phosphoryloxy-N,N-diethyltryptamine) (37), which were used in psycholytic therapy [e.g. (34, 38, 39)]. In the 1990s, Leuner also worked with the short-acting mescaline derivative 2C-D (2,5-dimethoxy-4-methylphenethylamine) (Figure 1).

In psycholytic therapy, dosages were individually adjusted so that the patient remained oriented to the present situation and in communication with the therapist. This preservation of a “reflecting ego remnant” (Leuner) marks a significant distinction between psycholytic and psychedelic therapy, where the patient may lose track of consensual reality for a period of time [e.g. (25, 40, 41)].

## Indications and contraindications

In an international discussion at the 1969 symposium of the *EPT* in Würzburg, Germany, a “list of indications” was assembled (cf. Table 2). The list includes a broad spectrum of diagnoses, using the language of the time, that were treated with psycholytic therapy.

**TABLE 2:** Main indications of psycholytic therapy as issued by the European Medical Society of Psycholytic Therapy (EPT) (42).

**Psychopathological conditions**
Character neuroses
Psychopathic individuals
Obsessive-compulsive neuroses
Neurotic depression
Endo-reactive depression
Anxiety neuroses
Cardiac phobias or cardiac neurosis
Phobias
**Sexual disorders**
Chronic impotence and frigidity
Exhibitionism
**Psychosomatic conditions**
Migraine
Psoriasis
Ulcerative colitis
Pubertal anorexia
Hysterical conversion

The terms used in the table - admittedly outdated in terms of diagnostic designations - are intended to present a historical reference and starting point for psycholytic psychotherapy. This is also in view of the fact that not much has happened since then in terms of research in psycholytic therapy and that future clinical trials may test the historical indications using rigorous study designs.

While many of these diagnoses are antiquated in their terminology, the human conditions they describe are certainly still present among patients seeking help from psychotherapy and substance-assisted therapy in general. Leuner repeatedly asserted that the indication for psycholytic therapy - as always in psychotherapy - “does not [depend] solely on the diagnosis of a case. Much more decisive are prognostic considerations that arise from the peculiarity of the patient … his sociological situation, the duration of the symptoms, and other factors” (42). Prognostically favorable, in his opinion, were: sufficient ego strength, success in professional life, suffering under genuine pressure of daily life, and intrinsic motivation for therapy.

*Absolute contraindications* were brain-organic conditions, serious liver and kidney diseases, endogenous psychoses, pregnancy. *Relative contraindications* were: lack of motivation for psychotherapy, histrionic personalities with tendency to act out, and personality disorders with lack of suffering. Most authors in the 1960s excluded patients with first-degree relatives suffering from psychosis for reasons of safety [e.g. (34, 40)].

## The method of psycholytic therapy

**FIGURE 2:**
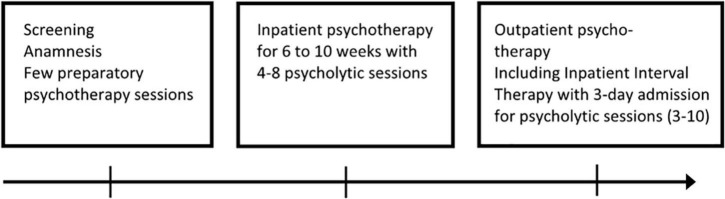
Overview of the psycholytic therapy treatment process.

Leuner and colleagues followed a somewhat flexible, but standardized treatment protocol (see Figure 2 and Table 3 for illustration): at the beginning of the treatment, questions regarding psychotherapy, substances, dosages and the setting to be used were discussed in detail with the patient in advance of the psycholytic sessions. This was followed by several interviews to learn about the patient’s life story and his medical history, to establish a working alliance and to prepare the patient for a psychodynamic framework for the substance session. Transference reactions were also a focus of the discussion. The preparatory sessions lasted 60 to 90 min and were conducted by one or two lead therapists.

**TABLE 3:** Flowchart of psycholytic therapy on the treatment day.

Timetable	Therapeutic activity
7.30 am	Light breakfast
8.00 am	Preparatory talk ⚬ Discussion of the content of the last session and time after last session ⚬ Discussion of dosage ⚬ Formation of a focus for the current session
8.30 am	Psycholytic session ⚬ Administration of the substance ⚬ Patient goes with sitter to treatment room ⚬ Putting on eye patches and playing soft background music ⚬ Lead therapist joins the patient at one-hour intervals
1 to 2 pm	Lunch
2 to 4 pm	Group therapy session
4 to 6 pm	Art therapy in the group
6 to 9.30 pm	Dinner and free time for oneself or with other patients
9.30 pm	Offering a light sleep aid
10 pm	Patients go to bed to sleep

Psycholytic therapy was typically initiated during an inpatient admission; such admissions usually lasted 6 to 10 weeks. 5 to 8 psycholytic LSD or psilocybin sessions were applied during this stay. A typical schedule on the day of the psycholytic session is outlined in Figure 2.

For the substance assisted therapy sessions, there typically was one support person (sitter) permanently in the room with the individual patient and one lead therapist who was in charge of the psychotherapeutic interventions and who was in and out of the room. The 4 to 6 patients scheduled for treatment on a specific day met in the morning for individual preparatory sessions with their lead therapists and the sitter. The selected therapeutic substance was usually given around 9 a.m. The duration of the drug-session was between 4 to 8 h, depending on the substance used. Immediately after the session, the patients and the sitters as well as the lead therapists met in a group therapy session to discuss the experiences. Thereafter, an art therapy group session was held where the patients were invited to give their subjective experiences and artistic expression by painting and working with clay.

Following the inpatient phase of treatment, the patient was usually transferred to a so-called Inpatient Interval Treatment. In this, the patient visited the clinic for a day and a night at 14-days (or longer) intervals for a psycholytic session.

### The environment

**FIGURE 3:**
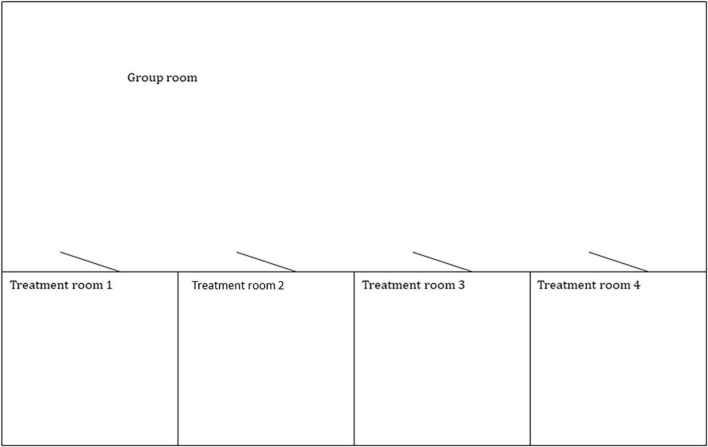
The illustration shows a treatment facility specific to psycholytic therapy as it was held in various clinics in the 1960s.

**FIGURE 4:**
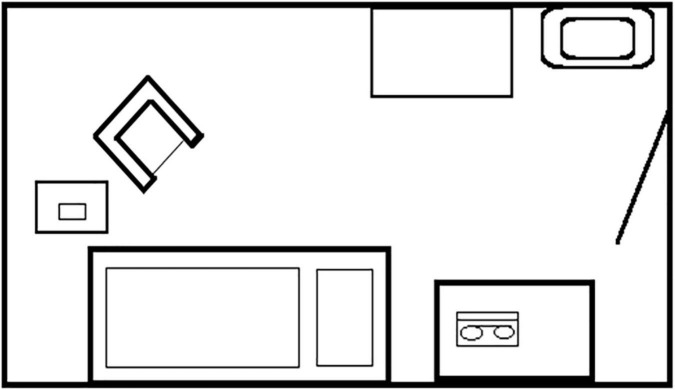
The illustration shows a treatment room with furnishings typically used in psycholytic therapy.

In a typical psycholytic clinic in the 1960s, there were several single rooms for the individual sessions and group rooms where group therapy and creative therapy took place after and between the psycholytic sessions. During the session, the patient was instructed to lie or sit on a bed. The room was simply but tastefully decorated. It was either soundproofed to minimize distracting outside noise or somewhat secluded from clinic noise (see Figures 3, 4).

### The staff

Psycholytic therapy was conducted under the direction of a psychiatrist who has been thoroughly trained in psychotherapy or of a doctoral level psychologist, similarly trained in psychotherapy. Supervision and case conferences were of considerable value. Sitters were instructed and called in for case conferences at regular intervals; some had supervised self-experiences to be more able to empathize with the patients. The sitter’s role was to establish an informal and relaxed atmosphere in which the patient felt comfortable and safe. Otherwise, sitters were expected to be flexible in adapting his or her responses to the situation. However, the requirements varied greatly. Some patients required only brief contact from time to time, while others desired assistance throughout the session. In general, more flexible empathic behaviors were required than in ordinary psychotherapy sessions. Active support was sometimes necessary by a question or an offer to talk, but the basic attitude of the sitters was one of quiet presence and containment. Non-invasive physical touch (e.g., holding a hand) was offered, provided careful informed consent was obtained during the preparatory session. Interestingly, every psycholytic therapist in the past was in favor of marginal physical touch (e.g., holding a hand) as an occasionally necessary component of psycholytic therapy [e.g. (34, 35, 43, 44)]. Leuner’s rule of thumb was to limit therapeutic touch to the region upwards of the shoulder, including arm and hand (45).

In individual cases, elements of Psychodrama were useful to bring into the session. In this process, the patient, as the main actor in the here and now, created his therapeutic theme as if on a dramatic stage, in accordance with Psychodrama procedures (Moreno, etc.). The therapist allowed himself to be touched by the patient’s play as a co-player or spectator, intervened if necessary, and then gave feedback. This approach could lead to a healing experience of abreaction or catharsis. The goal was for the patient to find a new and appropriate reaction to a new situation or one that was already well known.

The attitude of the lead therapist was one of calmness and empathy, striving to create a positively valenced, secure and safe setting. Therapists had well honed skills in perceiving the patient’s emotional transference and their own countertransference reactions (41, 45). According to *EPT* guidelines, it is highly valuable for the therapist to have had at least 5 supervised self-experiences to deepen the understanding of the spectrum of inner experiences and to guide the patient with a maximum of empathic attunement.

### The patients

Patients were invited to assume a relaxed position and asked to pay introspective attention. They were asked to surrender themselves openly to the impressions, emotions and visions which may appear. Verbal expressions by the patients were recorded. These recordings were used in integration sessions following the psycholytic sessions. The psycholytic sessions evoked an intense emergence of psychodynamic contents of the patients; there may or may not has been overt connection to explicit symptoms. It this way, psycholytic therapy is a particularly powerful tool to awaken imagination and awareness of conflicts, and honesty about the self (46).

Therapist and support person must ultimately follow a middle course in order to provide the patients with a sense of security on the one hand and avoiding intrusiveness or an excess of guidance that is based on the therapists sense of what should be happening, rather than the patient’s. A flexible, intuitive balance between the two emerges with practice. Some patients need this human contact more than others. In both the time of Hanscarl Leuner’s work and present psycholytic therapy it is the standard approach to not try to stop or decrease abreactive patterns, but instead, to support the expression of intense emotions, communicating the value of the intense experience. The patients may desire to talk, but cognitive discussions, practical questions or small talk should be avoided. The patients are in a heightened state of sensation, sensitive to small things, and may become irritated or distraught easily. The therapy environment may be full of projections, fantasies, longing for love or frantic despair. All these are held as welcome phenomena by the therapists and caregivers in the room. Training in being the recipient of erotic or paranoid transferences is vital for therapists in this work.

### The accompanying psychotherapy

The psycholytic sessions were generally embedded in a long-term psychotherapy. Psycholytic therapy done by Leuner and colleagues during the 1960s had 50 to 200 hours of psychodynamic or psychoanalytic therapy, with 5 to 25 psycholytic sessions interspersed in the treatment.

Leuner’s approach was congruent to the approach used today: both tend to have a more phenomenological intention: facilitating a narrative by the patients of their experience, saving any interpretation or meaning making for later. As the peak intoxication period ends, and usually following a period of high affective arousal, an opportunity for verbal exploratory work comes, and this period is noted for its significance by historical and contemporary practitioners alike. During the fading intoxication the patients still experience a decrease in defensiveness, the expansion of associations and an altered feeling of connection with the therapist, the sitter and other patients. The psychodynamic group therapy session immediately after the psycholytic sessions is a particularly rich setting for quiet sharing, story telling, relational processing, new perspectives and self awareness. The patients may be newly aware of resistances, defenses and fixed, rigid beliefs that need to be released.

Following the group session, an art room setting is provided for the group for possible growth experiences using drawing, painting, writing or other play. Thereby, non-verbal experiences, realizations, metaphors of transformation are supported.

During the following days, each patient has individual sessions with his therapist, and usually transcripts (based on the recordings of the psycholytic sessions) are introduced here. One method would be to apply a therapist’s usual way of working with dreams to the psycholytic journey. The degree of active interpreting versus evocation of narrative telling will vary among different therapists and their way of working.

### Psycholytic group therapy

With psycholytic therapy, in Leuner’s days, primarily the individual model was used for conducting psycholytic sessions. Experiments with conventional group therapy settings during the medicine session were abandoned because of the inability to follow-up group interactions, e.g., by distractions from individual absorption (“tripping”). Sandison and Leuner put group therapy in the period immediately after the psycholytic sessions to use the loosening of defenses in the post intoxication phase for psychotherapeutic work.

Some psycholytic therapists experimented with modified group treatments with some success [e.g. (47–49)], but psycholytic group therapy never became broadly used [cf. (50)]. However, it experienced a revival with the Swiss Physicians Society for Psycholytic Therapy (SÄPT) since the late 1980s [cf. (36, 51)].

### Dose and number of sessions

The substance and the dose should be chosen very carefully. One goal is for the person to remain fully oriented and capable of communication with the therapist; this includes, of course, the full awareness of the specific therapeutic situation that is happening. Leuner has referred to this as “double consciousness” and named it technically as the “reflective ego remnant” (34).

Dosages used in Leuner’s days for psycholytic therapy were in the lower range of usually 75-125 μg LSD (range 50-200 μg) or 8-15mg psilocybin by mouth. According to a psycholytic discourse, the dose cannot be determined linearly in relation to body weight, but preferably should be based on the patient’s characteristics (e.g., constitutional factors, obsessive vs. hysterical personality structure, specific defense mechanisms). Obviously, these attributions are embedded in the doctors training and culture, as well as countertransference reactions evoked by the patient. In the 1950’s, Sandoz, as well as the EPT guidelines recommended gradually ascending dosing of, e.g., LSD in increments of 10-25 μg per session until the desired level of consciousness alteration was achieved. The criteria for the correct dosage were a noticeable psychic activation, emergence of some psychedelic phenomena but not all, and the preservation of the reflective ego capacity. If the latter was lost, the dose was considered too high.

Usually, one session was given every two to three weeks, with 1-3 psychotherapeutic hours per week in between. This procedure was modified according to the individual patient and his progress. About 5-10 psycholytic sessions were used for mild cases, and about 20 sessions on average for severe cases [e.g. (41, 42, 52)].

### Psychological phenomena in psycholytic therapy

Since psycholytic therapy does not aim at the “dissolution of the ego” as is the goal in psychedelic therapy, some specific psychic phenomena emerge in psycholytic sessions.

Leuner offered a cartography of each LSD session having three phases that can be distinguished (25).

### First phase (hours 1-3)

A daydream-like twilight develops with elements of altered feeling and mood, but little verbal communication. Unusual perception of the environment is common, including hyperawareness of somatic sensations. Biographically determined pictorial-symbolic representations of personal problems strongly preoccupy the patient. Regressions into non-verbal states can occur, but less than is seen in psychedelic therapy. The therapist’s overt attitude is of promoting a contemplative attitude for “introspection.”

### Second phase (hours 3-4)

This phase usually contains a stronger attention to the therapist; intensified transference phenomena are seen (this may evoke countertransference phenomena in the therapists). Commonly seen is an increased willingness to communicate (talkativeness, garrulousness) and self-reflection. It is generally recommended to leave the self-oriented narratives to the patient and refrain from creative interpretations at this time.

### Third phase (hours 5-8)

In this phase, there is often a pronounced wish to communicate verbally. Experiences are described in detail. Curiosity and discovery are the therapist’s goals, not formulation or problem solving, even though the therapists may feel moved to make such formulations. Deeply meaningful insights into one’s own fixed beliefs and relationship patterns may become extraordinarily apparent to the patient.

In addition, LSD has been described by Fernandez-Cerdeno and Leuner (53) and others [e.g. (54)] as leading to the re-experiencing of repressed childhood memories at a somatically real level, so that the patient feels as if he were at the age in which the traumatic experience took place (so-called age regression). This may be accompanied by high levels of somatic arousal.

Unlike psychedelic therapy, psycholytic therapy aims at a less deeply altered state, with less loss of core self identity. It does not aim at a dissolution of the ego, but only at a temporary change or softening of ego experience. This serves to facilitate access to repressed conflicts, affects, fantasies, and memories in a psychological state, which allows a familiar process of psychotherapeutic work, including emotional abreaction, insightfulness, and compassion that the therapist can support.

Another core concept derived from Leuners basic research: Leuner, in 1962, based on observations from more than 1,000 LSD sessions, described two typical courses the experience can take (25). These are: (1). the continuous-scenic course and (2). the stagnating-fragmentary course. We offer these as one possible cartography of the psycholytic process. Which of the two courses occurs is partially determined by the dose level i.e., an individually adjusted dosage can influence which kind of course will occur.

### The continuous-scenic course

This is characterized by scenic imaginations with closed eyes, which develop in coherent processes - occasionally like the scenes of a film - and are closely integrated with affective resonance. Sense-filled emotional experience flows in a continuous stream of consciousness. The contents and scenes are mental projection processes, sometimes described as complex imagination, and show individual biographical meaning-relatedness. Age regressions may occur, as can vivid childhood memories. The experience has a continuous sequence and often stimulates the person to meaningful reactions and reflections. The reflective ego capacity is preserved and the patient can face, describe and reflect on the experiences with some distance.

### The stagnating-fragmentary course

In this course form, the experience may show some features of the scenic course, but instead of the fluctuating emergence of the contents, a stagnation of the contents occurs. Over longer periods, the person may be repeatedly occupied by the repetitive emotional memory and complex visions with painful emotional content. The flow of experience seems “stuck” and the condition is experienced as one of torment. There may be an erratic quality and choppiness of inner sequences of experiences, similar to psychotic disjointedness and disconnection. The complex perceptions are formed as single elements, or fragments of whole objects; there may be fragments of objects or human-animal figures. As single, disconnected parts they stand next to each other but in a non-integrated way, and follow one another as isolated, unconnected single images. The individual perceptual contents become sluggish and viscous, separated by pauses, and a sense of fragmentation. An integrative context is no longer recognizable. Frequently, emotions without subject matter or content become interspersed in the experience. The patient’s reflective ego capacity may be missing. Arousal-consuming regressive symptoms such as psychomotor phenomena, restlessness, motility disorders, or instinctual actions may be seen.

Leuner and others at that time considered only the continuous-scenic course as therapeutically beneficial. Therefore, by carefully considering the dosage and the therapeutic container, the conditions for the continuous-scenic journey are offered. The main sign that the dosing was correct is that the reflective capacity of the ego is preserved.

### Transphenomenal dynamic control systems

Leuner hypothesized that many LSD phenomena observable in the context of psycholytic therapy can be understood through a concept of specific *memory constellations.* These constellations selectively determine the field of experience. Such systems controlling the sequence of experiential contents were described by Leuner (25), who named them “Transphenomenal Dynamic Control Systems” (tdySt). They are “transphenomenal” in the sense that they are not restricted to specific mental functions or any particular life event. Rather, they propagate through the personality in various functions of thought and behavior. Leuner [(25), p. 119] defines these constellations as “driving emotional forces of a tense dynamic system, an unfinished need pressing for completion or settlement.” Not every unfulfilled need becomes an experiential phenomenon “rather, the symbolic image enters consciousness as a substitute, as it were as a preceding part, as an image projection of this emotional state. The symbolically representing emotion, more generally speaking dynamics, remains transphenomenal.” [(25), p. 119]. In the context of psycholytic therapy, LSD “thereby assumes the role of an activator of the affective complexes which had hitherto been dormant or, in any case, had expressed themselves only in a relatively lavish and symptomatic manner” [(24), p. 97]. Some activated tdySt can be integrated on a verbal level, others on a symbolic level, others at a somatic level, and others through the disclosure of a traumatic event and its psychotherapeutic processing.

Leuner’s tdySt shows extensive congruence with the Systems of Condensed Experience (COEX-Systems) postulated later by LSD therapist Stanislav Grof (54) from his early work with psycholytic therapy. Grof defines a COEX-System as a specific constellation of memories (and/or fantasies) from different stages of a person’s life. The memories belonging to a particular COEX-System have a similar underlying theme or common basic elements and are occupied by strong emotions of the same quality. The deepest layers of these systems usually contain very vivid, colorful memories of early childhood experiences. The more superficial layers store memories of similar experiences from later in life to the present life situation. The emotional intensity of the COEX-Systems (recognizable by violent abreactions accompanying the uncovering of these systems in LSD sessions) seems to be a summation of all emotions belonging to the memories of a particular system. As a rule, after activation of a COEX-System under the influence of LSD, there is reliving of related memories as well as cathartic abreactions, which then leads to their integration, also via the accompanying psychotherapy. Afterward, the COEX-System in question loses its energetic charge and thus its influence on the field of experience and the psychological state. Repeated LSD sessions can thus be viewed as successive uncovering, abreaction, and integration of individual COEX-Systems. However, like Leuner (25) and Grof (54), psycholytic therapists Ling and Buckman (41) point out that abreaction alone is not of lasting value unless the associated feelings are processed and integrated by the patient in a psychotherapeutic framework.

## Mechanisms of action in psycholytic therapy

The psychological mechanisms of action of psycholytic therapy were concisely described by Abramson (55), Grof (54, 56), and Leuner (25, 42).

• Activation of affect and sensory perception
• Loosening of psychological defenses
• Personally meaningful symbolic visualization of affects
• Therapeutic splitting of ego functions with a preserved “reflective ego capacity”
• Broadening of associations
• Exceptionally vivid reliving of disavowed conflicts and memories
• Age regression with great depth of experience
• Pronounced affective abreactions
• Intensification of transference experienced and observable in the here and now on the therapist
• Spontaneous introspective insights

As these authors consistently described some psychological mechanisms based on systematic clinical observations of more than a thousand cases, it is likely that their observations remain resonant and valid.

In addition, there is the increased possibility of *corrective new experiences* in both the intrapsychic and especially the interpersonal areas. This has important implications in relation to Grawe’s postulated “neurobiological reshaping through re-experience” (57). “The *clarification perspective*, which in the form of improved introspection and insight into the psychogenesis of the disorders and problems, into the roots of one’s own life history, but also into one’s creative potentials as well as into one’s own possibilities of experience and behavior, has a great significance in psycholytic treatment” [(58), p. 74].

## Possible risks and complications

Two major surveys in 1960 and 1971 covering psycholytic (and psychedelic) applications with more than 9,000 patients and a more recent retrospective survey from Switzerland (59–61) show that the risk of major complications (suicide attempts, suicide, prolonged psychotic reactions) is not higher for patients in psycholytic therapy than those in conventional psychotherapy (Table 4).

**TABLE 4:** Suicide attempts, suicide and prolonged psychotic reactions in psycholytic/psychedelic therapy.

Survey	Patients	Suicide attempts	Suicide	Prolonged psychotic reactions
Cohen (60)	ca. 5,000	1.2:1,000	0.4:1,000	1.6:1,000
Malleson (61)	ca. 4,300	0.7:1,000	0.3:1,000	0.9:1,000
Gasser (59)	121	0	0	0

## Studies on the efficacy of psycholytic therapy

Because of the long interruption of research and a certain neglect during the last thirty years of the “psychedelic renaissance,” studies of psycholytic therapy are scarce. This was, among other things, due to the unsophisticated and lax methodology used for psychotherapy research at the time. It was in its infancy and without the mature methodology that would emerge in contemporary academic settings. Mascher published a review in 1967 [(62), pp. 441-444] about results as well as favorable indications for psycholytic therapy. Regarding treatments rated as “successful,” psycholytic therapists reported long-term improvement in approximately two-thirds of their difficult to treat and chronic psychoneurotic patients (see Table 5).

**TABLE 5:** Treatment results for different groups of treatment-resistant patients with psycholytic therapy (62).

Diagnoses	Number of studies	Treatment success rates
Anxiety disorders	9	70%
Depressive neuroses	4	62%
Personality disorder	10	61%
Sexual disorders	7	50%
Obsessive-compulsive disorder	7	42%
Hysteria and conversion	2	31%

Due to the marginalization of psycholytic therapy and psychedelic therapies in general in the late 1960s, larger controlled studies could not be conducted. Two research projects were conducted by Leuner, with the goal of studying the efficacy of psycholytic therapy. These were naturalistic studies. As mentioned above, practitioners of psycholytic therapy were focusing on conditions that were not helped by conventional therapy. In these refractory patients, it was reasoned, virtually all improvements achieved would be considered a success, and an analysis of pre/post outcomes could accordingly be taken as evidence of efficacy. With this in mind, Leuner’s research group followed up patients treated at the Göttingen clinic (Germany) from 1959 to 1985 (63, 64). Diagnoses of patients were: anxiety neuroses and phobias, cardiac neuroses, personality disorders, conversion neurosis, neurotic and reactive depression, and sexual deviations. Both studies focused on performance in everyday life and at work, using an established grading to characterize the patients general status. Number of psycholytic sessions varied between 5 and 25, with an average of 12. The two follow-up studies - with obvious limitations - demonstrated a good success rate of more than 66% and a favorable economic profile. If these results could be confirmed in controlled studies, psycholytic therapy, especially when applied with treatment-refractory patients, could make a significant contribution to psychotherapeutic care.

## Discussion

After a hiatus of about forty years since the United Nations’ prohibition of psychedelic substances in 1971, therapeutic research resumed some 30 years ago. However, there seems to be a recent dominance of psychedelic therapy and a neglect of psycholytic therapy. This is somewhat surprising, given that less than one third of the publications about hallucinogen-assisted psychotherapy in the 1955 to 2000 timeframe had been about psychedelic therapy, the other two-thirds about psycholytic therapy [cf. (33)]. For various reasons, the psycholytic approach hasn’t been developed or cultivated in North America and is virtually unknown to most researchers and therapists today. The distribution of psycholytic therapy with more than 30 treatment centers and around 100 outpatient psychotherapists was much broader than that of psychedelic therapy.

One might ask whether the recent dominance of psychedelic peak therapy is an historical artifact. For instance, research on hallucinogens – which is very costly because of immense regulatory hurdles – has been mainly financed by non-commercial philanthropy; wealthy U.S. individuals, during the 1990 to 2010 timeframe, showed a pattern of financial support unknown in Continental Europe. In this context, because of its short-term application, the psychedelic approach has a clear advantage over the psycholytic approach. Studies are easier to design and conduct, which makes efficacy studies less expensive than long-term psycholytic therapy research. This is true not only for the comparison between the psycholytic and psychedelic approach, but also for longer-term versus short-term psychotherapies, where evidence has been in favor of longer-term psychotherapies, especially in complex and chronic cases (65–69). A relevant background of the recent enthusiasm regarding the therapeutic use of hallucinogens and entactogens is the fact that after 30 years of neurobiological research in psychiatry, no breakthroughs in respect to new pharmacological agents has been reached (70). This created significant “therapeutic underpressure” in psychiatry for new approaches. Against the backdrop of this underpressure, anything that appears to be effective is readily embraced, though often with heightened expectations.

The mystical experience of unity and connectedness is central in psychedelic therapy and has been described as its most important active factor (71, 72). Although it seems that mystical experiences are correlated with symptom reduction in certain groups of patients, some controlled studies in the past as well as today have failed to prove a causal relationship [e.g. (73)] or rely on largely uncontrolled trials (74).

Mystical-type experiences have been observed by psycholytic therapists as well. However, in psycholytic therapy, mystical-type experiences are not a specific goal, given that retaining a state of self-reference is a central characteristic. Nevertheless, the psycholytic approach might also be suited for treatment of life-threatening diseases given that this specific group of patients usually does not suffer as much from entrenched long-term neurotic dysfunctional behavioral, relationship and psychosocial performance. This could imply that their psychological coping resources are more intact and salutogenetic forces easier to access and activate. In this vein, the activation of self-healing forces has been discussed as an essential part of psycholytic, psychedelic and MDMA-assisted therapies likewise [e.g. (75)]. The activation of self-healing forces could even represent a non-specific component for psychedelic-assisted psychotherapy, independent of the respective specific psychotherapy model.

MDMA-assisted psychotherapy differs from psychedelic therapy, since MDMA does not induce an ego-dissolution with mystical overtones (76). In MDMA-assisted psychotherapy, ego functions and cognitive functions are preserved to a large degree. Therefore, MDMA-assisted psychotherapy shares many similarities to the psycholytic approach. MDMA-assisted psychotherapy has been used in psycholytic contexts in Switzerland in the 1988 to 1993 timeframe as well as today in a compassionate use program [cf. (77)].

In contrast to the psychedelic model, the psycholytic model interprets mystical-type experience not primarily as a transpersonal experience. Some psycholytic therapists argued that their patients tend to use these experiences to avoid working on their real everyday problems [e.g. (78)]. However, they recognized the healing power of such aspects of “synthetic-magical integrative experiences,” as they sometimes called them. They’ve found them often occurring at the end of psycholytic therapies and described them as a reinforcing therapeutic progress.

It is interesting to know that the group of researchers at the Maryland Psychiatric Research Center - the most prominent center for psychedelic therapy worldwide (79) - concluded, in view of their inconclusive study results [e.g. (3, 8)] that a combination of psychedelic and psycholytic therapy would be an interesting option, especially to enhance durability of treatment results. In this vein, Grof (80) and Yensen (81) proposed to name that combined approach “psychodelytic therapy”. Eventually, this group conducted several studies using the psychodelytic approach with neurotic outpatients and inpatient alcohol dependent patients by administering compounds with a shorter duration of action such as dipropyltryptamine and psilocybin (82, 83). In respect to the treatment of neurotic patients or those with personality disorders, “there was a suggestion that the single or double dose approach was inadequate for this level of pathology” [(82), p.13].

In sum, it seems that the psycholytic approach, while being dominant in Europe in the 1960s, has been neglected in the recent revival of substance-assisted psychotherapy. However, MDMA-assisted psychotherapy does not follow the recent popular psychedelic approach of using the ego-dissolving mystical experience as a focus of therapeutic change. Similar to classic psycholytic therapy, MDMA-assisted psychotherapy aims to just gradually alter ego functions and defenses, and focuses on reprocessing of trauma, catharsis and corrective emotional experiences.

If, as in the best studies of the 1960s, it should again be seen that the successes of psychedelic peak therapy are not broadly effective for all patients [e.g. (84, 85)], or are not lasting for those patients, the currently postulated “psychedelic renaissance” (1) may well be followed by a “psycholytic renaissance.” This renaissance would presumably be more modest in its basic attitude and characterized by acknowledgement of the protracted and small-scale nature of psychotherapeutic change processes. In addition, psycholytic therapy described above as well as a combination of psychedelic and psycholytic paradigms warrants further investigation in the future.

## Author contributions

TP and RK developed the conception of the review. TP wrote the first draft of the manuscript. RK and JG wrote sections of the manuscript. All authors contributed to manuscript revision, read, and approved the submitted version.
